# Meditation-Induced Coherence and Crucial Events

**DOI:** 10.3389/fphys.2018.00626

**Published:** 2018-05-29

**Authors:** Rohisha Tuladhar, Gyanendra Bohara, Paolo Grigolini, Bruce J. West

**Affiliations:** ^1^Center for Nonlinear Science, University of North Texas, Denton, TX, United States; ^2^Information Sciences Directorate, Army Research Office, Research Triangle Park, NC, United States

**Keywords:** Yoga and Chi meditation, criticality, heart rate variability, coherence, cognition, stress reduction

## Abstract

In this paper we emphasize that 1/*f* noise has two different origins, one compatible with Laplace determinism and one determined by unpredictable crucial events. The dynamics of heartbeats, manifest as heart rate variability (HRV) time series, are determined by the joint action of these different memory sources with meditation turning the Laplace memory into a strongly coherent process while exerting an action on the crucial events favoring the transition from the condition of ideal 1/*f* noise to the Gaussian basin of attraction. This theoretical development affords a method of statistical analysis that establishes a quantitative approach to the evaluation of the stress reduction realized by the practice of Chi meditation and Kundalini Yoga.

## 1. Introduction

The phrase normal sinus rhythm is known to be an overly simplified characterization of the true rhythmic pattern made by the series of time intervals between successive human heartbeats (Peng et al., 1993). Instead of a steady regular time interval between beats, healthy hearts manifest a heart rate variability (HRV), whose time series has a rather broad frequency spectrum. Historically, the frequency spectrum was explained to be a consequence of the firing rate of the sinus node (the heart's natural pacemaker) being controlled by signals from the autonomic (involuntary) portion of the nervous system, which has two major branches: the parasympathetic (decreases firing rate) and the sympathetic (increases firing rate). It is this continuing tug-of-war on the sinus node, with one decreasing and the other increasing the firing rate of the sinus node pacemaker cells that produces the HRV spectrum in healthy individuals. A number of techniques have been used to establish that HRV time series are fractal, for example, see Ivanov et al. (1999); Stanley et al. (1999), and consequently the frequency spectrum scales, for a review see West (2013). Herein we present a picture of the source of the HRV spectrum based on the statistics of the HRV time series.

Stress is known to disrupt the normal HRV spectrum and learning how to circumvent the debilitating influence of stress is one of the purposes of meditation. The origin of meditation is unknown, but scholars agree that it has been part of civilized culture for thousands of years, with the earliest records, circa 1,500 BCE, involving Vedantism, a Hindu tradition in India. As mentioned by Chow (2015) the *Yoga Sutras of Patanjali*, outlining the eight limbs of yoga, was compiled between 400 and 100 BCE. Cotemporaniously, the *Bhagavad Gita* was written, which discusses the philosophy of yoga and meditation.

From a physiological perspective meditation constitutes a coupling of the functionality of the heart with that of the brain and has been explored and developed for millennia. However, we are only now beginning to apply the methods of science and data analysis to this brain-heart coupling. The main purpose of the present paper is to provide a measure of reduction in the level of stress provided by meditation, which is to quantify, through the statistical analysis of HRV time series before and during meditation, precisely how much stress is alleviated by control through meditation of the heart-brain coupling.

### 1.1. Statistics and memory

One of the most difficult issues confronting complexity science is the origin of 1/*f*-noise (Watkins, 2016, 2017). Is it an ergodic or a non-ergodic process? Watkins (2017) has recently pointed out that Mandelbrot, very well known for his generalization of ordinary diffusion, called *Fractional Brownian Motion* (FBM) (Mandelbrot and Van Ness, 1968), which has a physical origin compatible with stationarity and ergodicity, is also the author of papers (Mandelbrot, 1965, 1967) opening a bridge between the stationary and non-stationary condition. Actually, the revisitation of the work done by Mandelbrot in the 1963-1967 period of time leads to the creation of a connection with the Continuous Time Random Walk (CTRW) (Montroll and Weiss, 1965; Shlesinger, 2017). This is equivalent to establishing a connection with the subordination perspective (Sokolov, 2000; Thiel and Sokolov, 2017) and with the assumption that *crucial events* determine the observed dynamics. The crucial events are characterized by the main property that the time interval between the occurrence of consecutive crucial events is described by the waiting-time probability density function (PDF):

$$\begin{array}{r} \psi \left(\tau \right)\propto \frac{1}{\tau^{\mu }}, \end{array}$$

According to Watkins (2016, 2017) the non-ergodic fractional renewal model of Mandelbrot (1965, 1967) yields the inverse power law (IPL) spectrum as a function of the frequency *f*:

$$\begin{array}{r} S\left(f\right)\propto \frac{1}{f^{\gamma }}, \end{array}$$

with

$$\begin{array}{r} \gamma =3-\mu . \end{array}$$

The dynamics generating crucial events are renewal, which means that the sequence of waiting times {τ_*i*_} between succsive events are completely independent of one another. This important physical property is expressed by the mathematical property of the two-point correlation function:

$$\begin{array}{r} C\left(i,j\right)\equiv \frac{\langle \tau_{i}\tau_{j}\rangle }{\langle \tau^{2}\rangle }=\delta_{i,j}, \end{array}$$

where δ_*i, j*_ denotes the Kronecker function; = 1 for *i* = *j* and = 0 otherwise, in the ideal limit 〈τ〉^2^ << 〈τ^2^〉. In this paper we focus on *C*(1) defined as *C*(1) = *C*(*t* = |*i* − *j*| = 1).

The physical interpretation of this mathematical property is that the occurrence of a renewal crucial event generates a rejuvenation of the system. Consequently, the system evolves toward the occurrence of the next renewal event as if the occurrence of the earlier crucial event made the system brand new. The renewal property facilitates resolving the 1/*f*− paradox (Watkins, 2016, 2017). This paradox has been brilliantly discussed by Niemann et al. (2013) who noticed that for γ > 1(μ < 2) the spectrum *S*(*f*) is not integrable, thereby violating the integrability condition on which the Wiener-Khinchine theorem is based. This theorem is the mathematical foundation necessary for the evaluation of a spectrum. This theorem has been side-stepped by working with a time series of length *L* to obtain (Lukovic and Grigolini, 2008):

$$\begin{array}{r} S\left(f\right)\propto \frac{L^{2-\mu }}{f^{3-\mu }}. \end{array}$$

This result is based on the observation (Feller, 1971) that the rate *R*(*t*) of production of crucial events, all prepared at *t* = 0, is given by the IPL:

$$\begin{array}{r} R\left(t\right)\propto \frac{1}{t^{2-\mu }}. \end{array}$$

Actually, by idenifying *L* in Equation (5) with 1/*f*, we obtain *S*(*f*) = 1/*f*, which is interpreted as an indication that working with a time series of length *L* makes *f* = 1/*L* the smallest observed frequency, thereby settling the paradox with no need of truncating the frequency distribution, insofar as the empirical truncation is a dynamical effect of the experimental observation.

It is interesting to notice that the restriction of the IPL index to the interval 2 < μ < 3 makes *S*(*f*) integrable, thereby generating a sort of equivalence between this process and that of FBM. To make this equivalence explicit let us focus our attention on the Langevin equation:

$$\begin{array}{r} ẋ=\xi , \end{array}$$

where ξ(*t*) is a correlated fluctuation adopted to make the diffusion process *x* identical to FBM. The rate equation thereby provides a dynamical origin for FBM (Cakir et al., 2006; Tuladhar et al., 2017). This approach is, in fact, based on a rigorous Hamiltonian model for a Generalized Langevin Equation (GLE), which can be made equivalent to the generator of Fractional Gaussian Noise (FGN) used by Kou and Xie (2004) to derive a diffusive process equivalent to FBM.

FBM is by construction stationary throughout the whole time region. On the other hand, the crucial events responsible for the IPLs denoted by Equations (2) and (3) generate a process that is not stationary, but one that becomes stationary in the long-time limit. Let us imagine that the time interval between consecutive crucial events is filled with either ξ = 1 or ξ = −1, as determined by a coin-tossing prescription. When μ > 2 the waiting-time PDF is used to calculate the non-stationary correlation function <ξ(*t*_1_)ξ(*t*_2_)>, which becomes stationary in the long-time limit. In fact, the coin-tossing prescription makes the correlation function of age *t*_*a*_, < ξ (*t*_*a*_)ξ(*t*_*a*_ + τ) >, identical to the probability that no new event occurs up to the time τ after the occurrence of an event at time *t*_*a*_. The brand new survival probability is proportional to 1/τ^μ−1^, but for *t*_*a*_ → ∞ the survival probability becomes proportional to 1/τ^μ−2^. Although the spectrum of these fluctuations is identical to that of FBM, with scaling:

$$\begin{array}{r} H=\left(4-\mu \right)/2, \end{array}$$

in the FBM case the regression of the correlated noise ξ(*t*) to the origin is characterized by an exponential waiting-time distribution. In addition the corresponding function *C*(*i, j*) violates the renewal condition of Equation (4), that is, it is not a Kronecker delta function.

The correlation function of FGN, as well as, the correlation function of fluctuations generated by crucial events may be extremely slow, even if *S*(*f*) with γ < 1 does not require the settlement of any paradox, insofar as 1/*f*^γ^ is integrable. There is evidence that the FBM long-time memory may be realized in many complex systems, for instance, climate, hydrology and finance, see the excellent review paper (Graves et al., 2017) to oversee the importance of this form of long memory.

Note that the FBM memory can be derived from Mori's GLE (Mori, 1965), which, in turn, is derived from Hamiltonian dynamics by projecting from the evolution of the entire Universe down to the dynamic variables of interest. This projection determines the system's memory in the sense that the intensity of the correlation function Φ_ξ_(*t*) of a variable of interest tends to vanish for *t* → ∞, but the decay is sufficiently slow that the correlation function Φ_ξ_(*t*) is not integrable. This form of memory, therefore, is a generalization of the well known Laplace determinism (Laplace, 1951), according to which given the initial conditions for all the atoms in the Universe one would be able to predict with no error the state of the Universe at any time in the future.

Herein we refer to this form of memory, including Laplace determinism, as *Hamiltonian Memory*. The deterministic motion given by the rate equation:

$$\begin{array}{r} \frac{d\xi }{dt}=i\Omega \xi \end{array}$$

is referred to as Hamiltonian memory for real Ω.

By way of contrast we refer to the non-integrable correlation function generated by the crucial event fluctuations as *Crucial Memory*.

How can one distinguish between these two forms of memory? The cross-correlation function of Equation (4) plays an important role in answering this question. In fact, in the case of crucial event infinite memory *C*(1) = 0, whereas in the case of FBM memory *C*(1) ≠ 0.

Is it possible that in real systems both forms of memory coexist?

Research (Allegrini et al., 2002; Bohara et al., 2017) reveals that in the case of HRV time series both forms of memory do, in fact, coexist. Although this coexistence is to some extent as paradoxical as that of wave-particle dynamics in quantum mechanics, it is not quite as surprising in biology and in the field of physiological processes. In the case of brain dynamics the concept of nested frequencies (He et al., 2010) was adopted to establish a connection between the physiological wave-like nature and the action of crucial events that are revealed by the proper method of analysis (Allegrini et al., 2015). It has to be stressed that Peng et al. (1999) revealed that meditation has the effect of generating a surprising coherent behavior in HRV time series. The connection between coherence and criticality is a subject of great interest (Termsaithong et al., 2012) that we address herein using the subordination perspective (Sokolov, 2000; Thiel and Sokolov, 2017). The connection between meditation and coherence is a subject studied by many authors moving from the adoption of positive emotions (Bradley et al., 2010) to different forms of meditation (Peng et al., 1999, 2004).

Herein we establish, with a statistical analysis of data using an approach developed in earlier work (Allegrini et al., 2002; Bohara et al., 2017) that the dynamics of heartbeats result in HRV time series hosting both Hamiltonian Memory and Crucial Memory. We also prove that meditation affects both kinds of memory, turning the Hamiltonian Memory into a coherent process (Peng et al., 1999). Meditation also has the surprising effect of affecting Crucial Memory, as well. In fact, our analysis shows that meditation shifts the IPL index μ of Equation (1) moving it from values very close to the perfect 1/*f* condition, μ = 2, to values near the border with the Gaussian basin of attraction, μ = 3. The meditation-induced coherence decreases the accuracy of the method adopted in earlier work (Allegrini et al., 2002; Bohara et al., 2017) to evaluate the percent of stress indicators, the Poisson events thought to be responsible for heart failure, but refine the methodology and establish that both Chi and Kundalini Yoga meditation have the effect of reducing stress, although the advanced practice of Kundalini Yoga meditation yields higher levels of reduction.

## 2. Subordination of harmonic oscillations

The subordination of a dynamic process to harmonic motion was studied by Ascolani et al. (2009). We assume that the clock's hour-hand completes a rotation from noon to noon through *M* clicks. The time interval between two consecutive clicks Δ*t* is normalized to 1. The frequency of the regular oscillator is given by:

$$\begin{array}{r} \Omega =\frac{2\pi }{M}. \end{array}$$

The regular oscillation is a deterministic Hamiltonian process equivalent to the process described by Equation (9). Being cognizant of the choice Δ*t* = 1, we set Ω ≪ 1 in order for the dynamics to be as close as possible to the coherent behavior of a regular clock. Each time interval between consecutive clicks is converted into an operational time τ, whose statistics are determined by the hyperbolic PDF:

$$\begin{array}{r} \psi \left(\tau \right)=\left(\mu -1\right)\frac{T^{\mu -1}}{{\left(\tau +T\right)}^{\mu }}, \end{array}$$

which is equivalent to realizing the time interval between crucial events being given by Equation (1). Numerically these statistics are produced (Lukovic and Grigolini, 2008) by drawing a random number *y* with a uniform PDF on the interval [0, 1] (0 < *y* < 1) and converting it into the operational time using:

$$\begin{array}{r} \tau =T\left[\frac{1}{y^{\frac{1}{\mu -1}}}-1\right]. \end{array}$$

**Figure 1:**
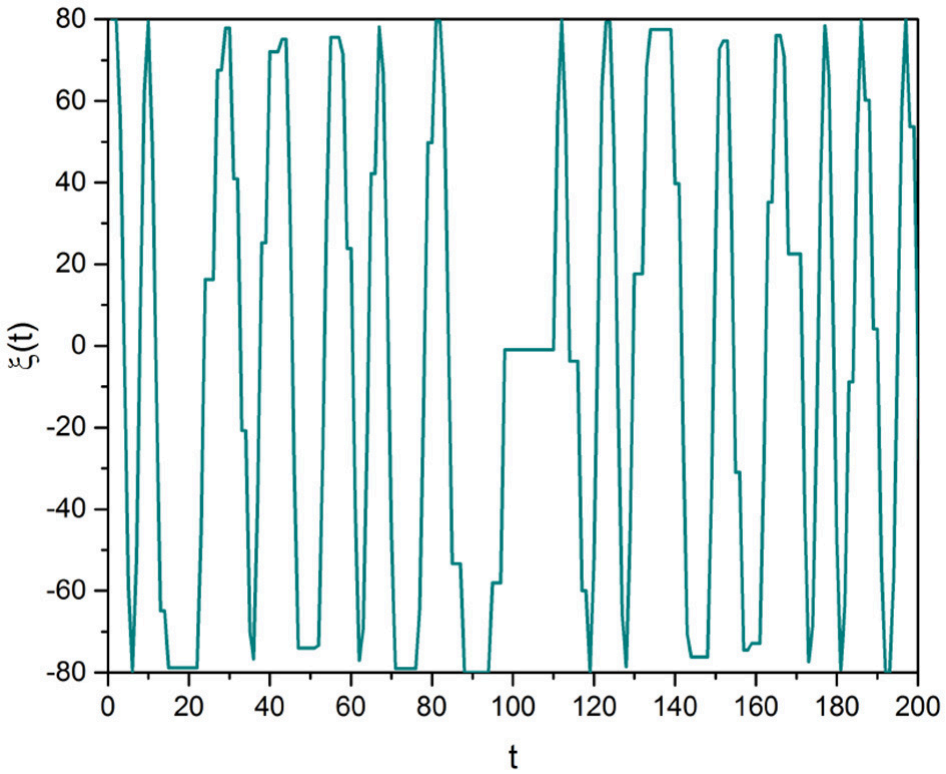
Subordinated cosine wave with Ω = 0.8 and μ = 2.8.

Each realization of this procedure yields a stochastic trajectory that for the clarity of discussion is here illustrated in Figure 1. Note that we focus our attention on μ > 2, a condition making the mean value 〈τ〉 finite, where the brackets denote an average taken using Equation (11):

$$\begin{array}{r} \langle \tau \rangle =\frac{T}{\left(\mu -2\right)}. \end{array}$$

The number of crucial events activated moving from noon to the long-time limit *t* is equal to *t*/ < τ >. Consequently, the number of cycles of the subordinated signal is equal to that of the coherent oscillator.

It is important to stress that, according the theoretical arguments using diffusion entropy analysis (DEA) (Allegrini et al., 2002; Bohara et al., 2017), in the long-time limit the crucial events generate a scaling process with index:

$$\begin{array}{r} \delta =\frac{1}{\mu -1}, \end{array}$$

which is numerically close to, but different from, the FBM scaling with an index *H* given by Equation (8).

The evaluation of the spectrum requires averaging the solution of Equation (9) over an ensemble of realizations. For this reason we define:

$$\begin{array}{r} Y\left(t\right)=Re\left[\langle \xi \left(t\right)\rangle \right], \end{array}$$

where ξ(*t*) is a single realization and the brackets denote an ensemble average. The subordinated process *Y*(*t*) can be derived from the following prescription (Ascolani et al., 2009):

$$\begin{array}{r} Y\left(t\right)=Re\left[\overset{\infty }{\underset{n=0}{\sum }}\overset{t}{\underset{0}{\int }}dt^{\prime }\psi_{n}\left(t^{\prime }\right)\Psi \left(t-t^{\prime }\right)e^{i\Omega n}\right], \end{array}$$

where ψ_*n*_(*t*) denotes the waiting-time PDF of a sequence of *n* events, the last of which occurs at time *t*, and Ψ(*t*) is the probability that no event occurs until time *t* far from the occurrence of the last earlier event. The evaluation of *Y*(*t*) is described in the theoretical work (Lambert et al., unpublished), which also affords rigorous mathematical arguments to evaluate the spectrum of this process. Herein we limit ourselves to adopting heuristic arguments supported by numerical results.

We evaluate the Laplace transform of the subordinated harmonic process *Y*(*t*), using the renewal character of the waiting-time PDF:

$$\begin{array}{r} {\hat{\psi }}_{n}\left(u\right)=\hat{\psi }{\left(u\right)}^{n}, \end{array}$$

where the Laplace transform of *f*(*t*) is denoted $\hat{f}\left(u\right).$ Inserting Equation (17) into the Laplace transform of Equation (16) and summing the series yields:

$$\begin{array}{r} \hat{Y}\left(u\right)=\frac{1}{u}Re\left[\frac{1-\hat{\psi }\left(u\right)}{1-exp\left(i\Omega \right)\hat{\psi }\left(u\right)}\right]. \end{array}$$

Note that in the long-time limit, namely for *u* → 0, this expression reduces to:

$$\begin{array}{r} \hat{Y}\left(u\right)=\frac{\hat{\Psi }\left(u\right)}{2}. \end{array}$$

Note that in the case where the waiting-time PDF is Poisson:

$$\begin{array}{r} \psi \left(t\right)=rexp\left(-rt\right), \end{array}$$

as shown by Ascolani et al. (2009), the subordinated harmonic solution obtained from the inverse Laplace transform of Equation (18), is:

$$\begin{array}{r} Re\left[Y\left(t\right)\right]=exp\left[-\left(1-cos\Omega \right)rt\right]cos\left[\sin\left(\Omega \right)rt\right]. \end{array}$$

The observation of the spectrum using real data suggests introducing the concept of an effective frequency ω_*eff*_ that, in the case of Poisson subordination, on the basis of Equation (21) reads:

$$\begin{array}{r} \omega_{eff}=r\Omega . \end{array}$$

**Figure 2:**
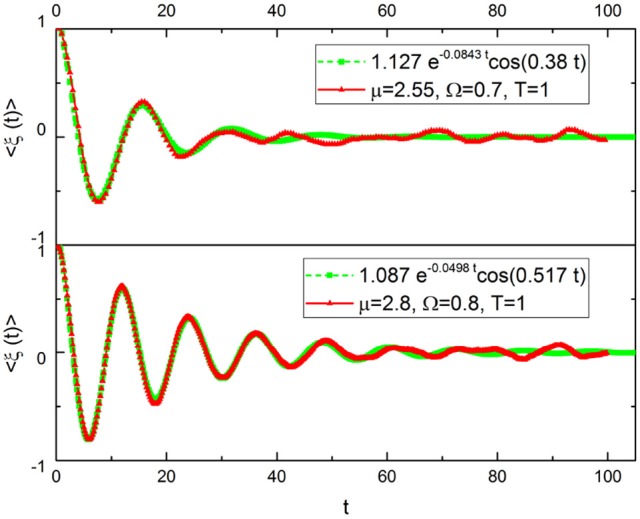
Ensemble average of ξ(*t*), μ = 2.55 **(top)** and μ = 2.8 **(bottom)**.

In Figure 2 we fit the numerical approach to subordination with the function:

$$\begin{array}{r} Y\left(t\right)=Ae^{-\lambda t}cos\left(\omega_{eff}t\right), \end{array}$$

using *A*, λ and ω_*eff*_ as fitting parameters, where we have used Ω << 1. Note that the analytical structure of Equation (23) is the same as Equation (21). The agreement is very good except in the long-time limit. The heuristic interpretation of this numerical result is that adopting μ > 2 generates an extended time region where the process is essentially Poisson. But in the long-time limit, as suggested by Equation (19), the survival probability Ψ(*t*) must appear. This explains the disagreement between the fitting and the numerical result in the long-time limit. The IPL index of the survival probaility Ψ(*t*) is μ − 1. This is a consequence of the fact that here we are considering the brand new survival probability. The spectrum evaluation done with very extended time sequences requires the aged version of *Y*(*t*) of Equation (23) which, in turn, in the long-time limit involves the aged survival probability with μ−2. This yields, as we shall see directly, Equations (2) and (3).

For the evaluation of the spectrum *S*(ω) for a time series of length *L* we adopt the prescription:

$$\begin{array}{r} \langle S\left(\omega \right)\rangle =\frac{1}{L}\langle |\overset{L}{\underset{0}{\int }}dte^{i\omega t}\xi \left(t\right){|}^{2}\rangle , \end{array}$$

which is equivalent to:

$$\begin{array}{r} \langle S\left(\omega \right)\rangle =2ReA, \end{array}$$

where

$$\begin{array}{r} A\equiv \frac{1}{L}\overset{L}{\underset{0}{\int }}dt_{1}\overset{t_{1}}{\underset{0}{\int }}dt_{2}e^{i\omega \left(t_{1}-t_{2}\right)}\langle \xi \left(t_{1}\right)\xi^{*}\left(t_{2}\right)\rangle . \end{array}$$

It is convenient to point out that the correlation function, under the integral in Equation (26), is not stationary, but does become stationary in the long-time limit. We expect that if the length *L* of the time series is sufficiently large, the spectrum defined by Equation (24) reduces to the ordinary Wiener-Khinchin prescription:

$$\begin{array}{r} S\left(\omega \right)=\frac{1}{2\pi }\overset{+\infty }{\underset{-\infty }{\int }}\Phi_{\xi }\left(t\right)exp\left(-i\omega t\right)dt, \end{array}$$

where Φ_ξ_(*t*) is the aged correlation function. The aged correlation function is a damped oscillatory function, with a tail characterized by the aged survival probability, namely an IPL with index μ − 2. We expect, therefore that for *f* → 0 the results of Equations (2) and (3) are recovered.

**Figure 3:**
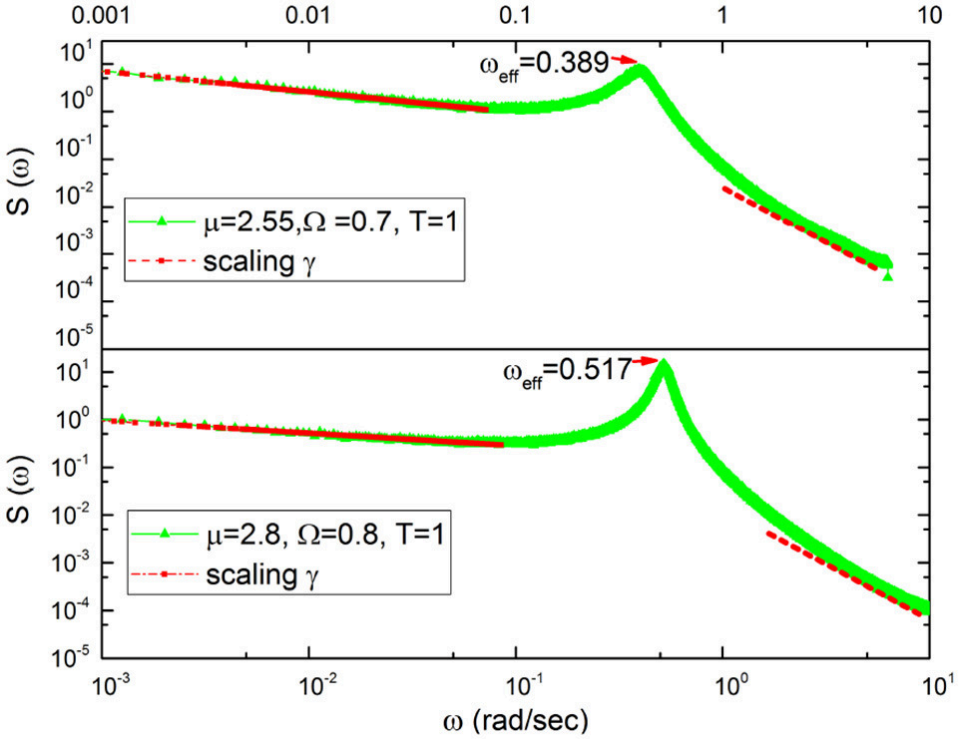
Spectra corresponding to μ = 2.55 **(top)** and μ = 2.8 **(bottom)**.The red lines are the fitting to the numerical results. **Top**: The red lines at the left of periodicity bumps yield γ = 0.43 to be compared to the theoretical prediction γ = 3 − μ = 0.45. The red lines at the right of the periodicity bump correspond to the prediction γ = 2. **Bottom**: The red lines at the left of periodicity bumps yield γ = 0.26 to be compared to the theoretical prediction γ = 3 − μ = 0.2. The red lines at the right of the periodicity bump correspond to the prediction γ = 2.

The numerical results of Figure 3 confirm our heuristic arguments. In fact, we see that the in the region of very small frequency the theoretical prediction γ = 3 − μ is satisfactorily confirmed. In the less precise case depicted on the bottom of the figure, we run μ = 2.8 and the numerical results correspond to an effective μ = 2.74. In the region of very large values of ω, the exponential decay of the damped oscillations generate γ = 2. Between the two IPL regions a bump appears, this being a clear signature of the effective frequency that becomes smaller by decreasing μ. This is qualitatively expected on the basis of the Poisson case of Equation (21). In fact, decreasing μ decreases the rate of event production, which corresponds to the effective frequency ω_*eff*_ = *r*Ω of Equation (21). Decreasing the rate of event production reduces the effective frequency.

## 3. Power spectra from real data

**Figure 4:**
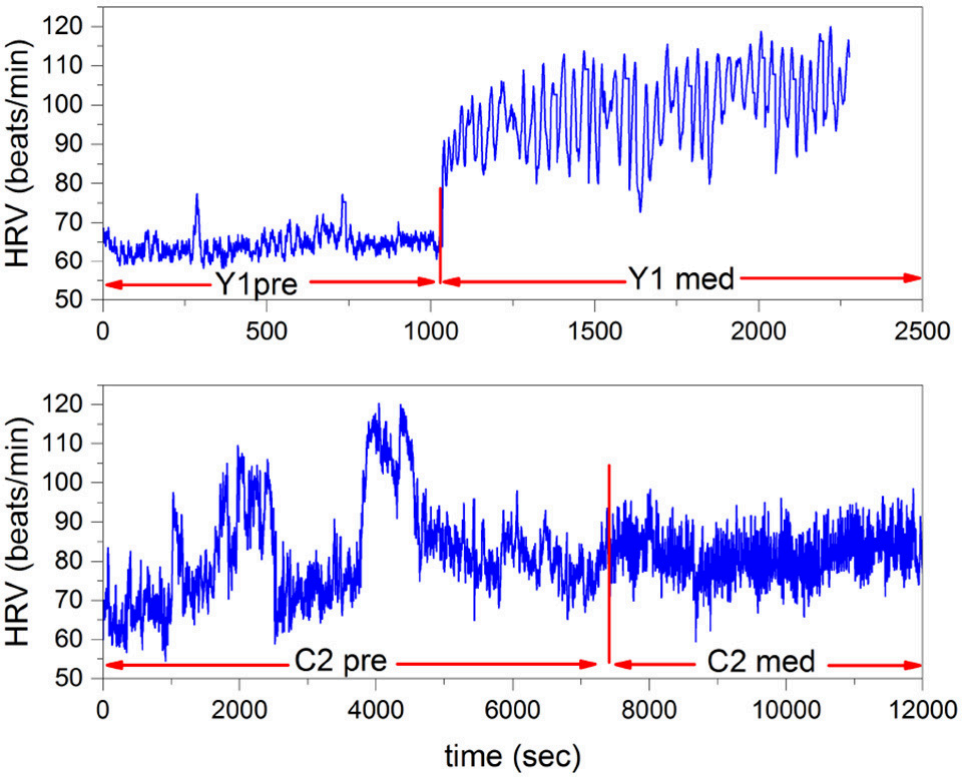
HRV time series of Yoga meditator (Y1), at the top, and the Chi meditator (C2), at the bottom. The vertical red lines denote the time at which the two meditations start.

In this section we illustrate the power spectrum of Equation (24) of two individuals, C1 and C2, practicing Chi meditation and two individuals, Y1 and Y2 practicing Kundalini Yoga mediation. To make our procedure clear in Figure 4 we depict samples of the HRV time series analyzed herein, referring to individuals Y1 and C2. The data is obtained from Physionet Goldberger et al. (2000), previously analyzed using very different techniques by Peng et al. (1999).

The Chi meditation is realized by instructing the subject to breath spontaneously while visualizing the opening and closing a perfect lotus in the stomach.

The Kundalini Yoga consists of a sequence of breathing and chanting exercises while seated in a cross-legged position.

In the experiment of Peng et al. (1999) the Chi meditators are novices and the Kundalini Yoga meditators are advanced practitioners. We see that the main qualitative difference between Kundalini Yoga and Chi meditation is that at the onset of medation the rate of heartbeats in the former case significantly increases, whereas in the latter it does not. However, during both types of mediation coherent-like oscillations appear.

**Figure 5:**
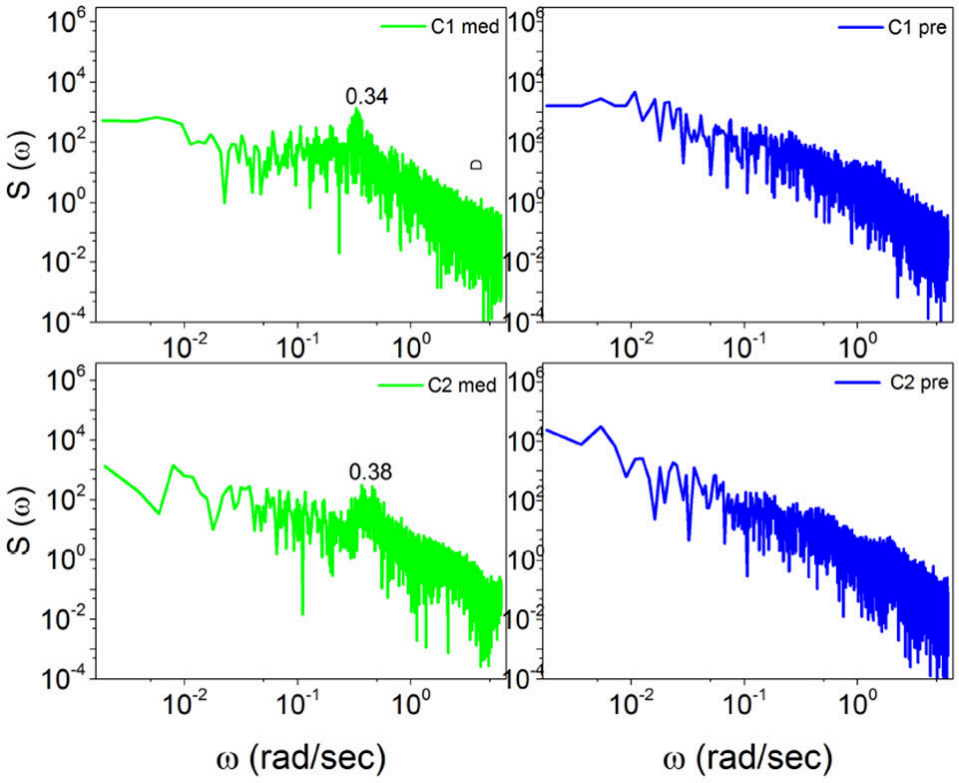
The power spectra *S*(ω) of Chi meditators (C1, C2) before, on the right, and during meditation, on the left. In the left panels, the spectra for C1 and C2 meditators consist of significant bumps at ω = 0.34(*rad*/*sec*) and ω = 0.38(*rad*/*sec*) respectively, each induced by meditation.

In Figure 5 the power spectrum *S*(ω) for the case of Chi mediation is depicted. We see that for both subjects, C1 and C2, the transition from the pre-meditation to the meditation condition induces significant periodicity bumps. Comparing the right to the left panels, a bump appears at ω ≈ 0.34 radians per second for C1 and at ω ≈ 0.38 radians per second for C2, each induced by meditation.

**Figure 6:**
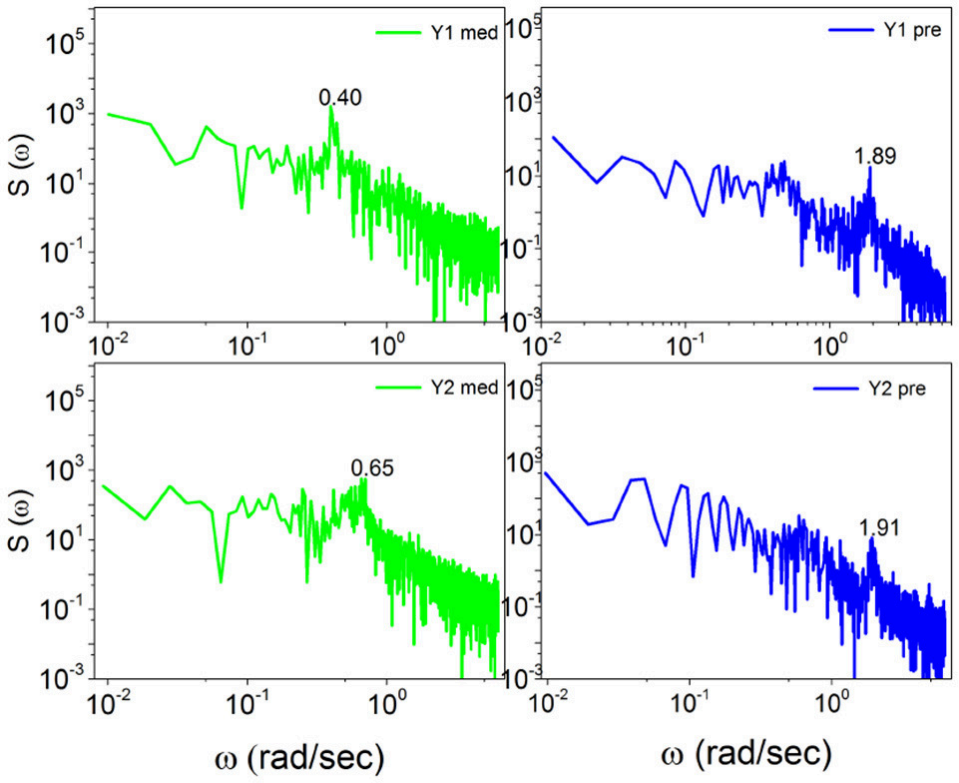
The power spectra *S*(ω) of Kundalini Yoga meditators (Y1, Y2) before, on the right, and during meditation, on the left. Notice the spectra for Y1 and Y2 meditators consist of significant bumps both before and during meditation. The bump shifts for Y1 meditator from ω = 1.89(*rad*/*s*) before meditation to ω = 0.4(*rad*/*s*) during meditation. Similarly the bump shifts for Y2 meditator from ω = 1.91(*rad*/*s*) before meditation to ω = 0.65(*rad*/*s*) during meditation.

Note that with the method of scaling evaluation presented in section 4 the IPL index μ of C1 is seen to change from 2.4, before meditation, to 2.55, during meditation. Similarly, the IPL index μ of C2 is observed to change from 2.28, before meditation, to 2.54, during meditation. Similar properties are found in the case of Kundalini Yoga meditation, Figure 6. In the latter case the periodicity bump during meditation for Y1 is ω ≈ 0.4 radians per second and for Y2 is ω ≈ 0.65 radians per second. Notice that there exists periodicity bumps for both Y1 and Y2 before meditation. These two subject are experienced practitioners and the fact that periodicity bumps exist, even before meditation, suggests that the practice of meditation yields permanent physiological effects. As far as the IPL index μ is concerned, the analysis done in section 4 shows that the individual *Y*1, before meditation, has the IPL index μ = 2.64, which moves to μ = 2.68. The individual *Y*2 has the IPL index μ = 2.66 before meditation and the IPL index μ = 2.72, during meditation. We shall discuss this shift more fully in section 5.

The similarity of the spectrum in Figure 6 with the idealized surrogate spectrum of Figure 3 is impressive. Actually, the top panel of Figure 6 has been realized using for the subordination process with the IPL index μ = 2.55, which is the mean value of the IPL index generated by Chi meditation and the bottom panel with the value μ = 2.8, which is the mean value generated by the Kundalini Yoga meditation. See section 4 for details.

We again note that the spectral analysis had been previously done by Peng et al. (1999), who were the first to find the meditation-induced bump. They did not, however, discuss the frequency region to the left of the bump, which according to our analysis is theoretically determined by crucial events. It is important to notice that Figure 3 is obtained by making averages using many numerical realizations. The information concerning the corresponding values of μ generating the spectrum to the left of the periodicity bump cannot be derived from the experimental spectrum. The determination of this information requires adopting the technique (Allegrini et al., 2002; Bohara et al., 2017) used by us in section 4.

## 4. Search of crucial events

As mentioned earlier, the search for crucial events is carried out using the method of stripes, developed earlier (Allegrini et al., 2002; Bohara et al., 2017). We observe the sequence of heartbeats recording the beat numbers in the HRV time series along the abscissa axis and the time interval between beats, *T*_*B*_(*i*), along the ordinate axis. The ordinate axis is divided into stripes of size *s* = Δ*T*_*B*_ ≈ 30 ms (Allegrini et al., 2002) and the crossing from one stripe to the nearest neighbor stripe is recorded as an event, but one that is not necessarily a crucial event. We expect, in fact, that the time interval between consecutive crucial events is filled with *pseudoevents*, as discussed for the surrogate data. Any event, either a pseudo-event or a crucial event, is assumed to make a random walker jump ahead by a fixed quantity equal to 1, thus generating a diffusion-like process. In spite of the fact that crucial events are rare, the long-time limit of this diffusion process is dominated by their scaling with index δ (Grigolini et al., 2001). Note that according to the scaling prediction of Equation (14), the IPL index is given by:

$$\begin{array}{r} \mu =1+\frac{1}{\delta }. \end{array}$$

The scaling index δ is evaluated numerically using Diffusion Entropy Analysis (DEA) (Grigolini et al., 2001). The pseudo-events imbedded between crucial events are characterized by Hamiltonian Memory, a form of memory additional to that of crucial events (Allegrini et al., 2002), as discussed earlier. The Hamiltonian Memory has the effect of violating the renewal condition *C*(1) = 0. Allegrini et al. (2002) assumed that the ideal condition of healthy heartbeats, with crucial events dressed with the additional memory of pseudo-events, yields *C*(1) ≈ 1. HRV time series also host totally random Poisson events, with probability 1 − ∈. Consequently, the evaluation of ∈^2^is done by evaluating *C*(1) of Equation (4) in the case of real sequences and setting the condition:

$$\begin{array}{r} \in^{2}=C\left(1\right). \end{array}$$

**Figure 7:**
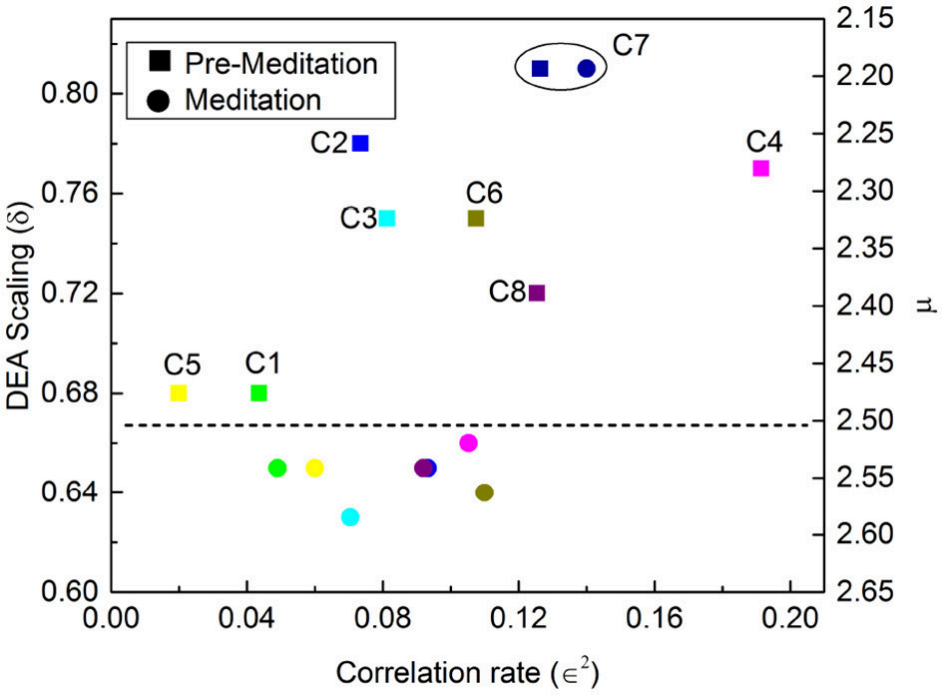
DEA scaling δ, IPL index μ and ∈^2^ of the HRV time series of eight different participants before and during Chi meditation.

**Figure 8:**
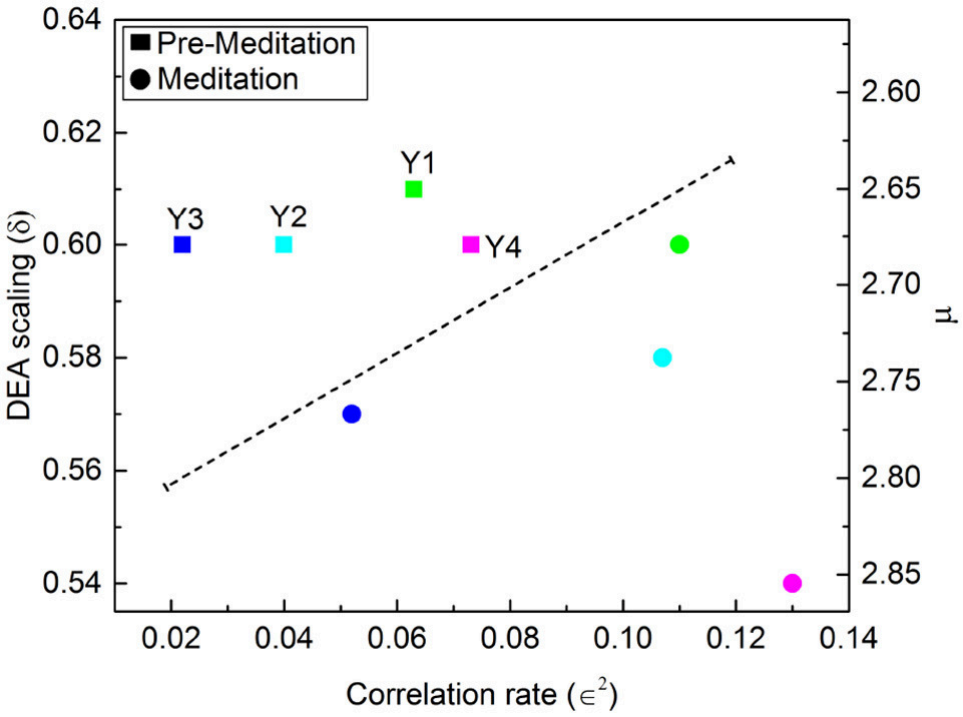
DEA scaling δ, IPL index μ and ∈^2^ of the HRV time series of four different participants before and during Kundalini Yoga meditation.

Figures 7, 8 illustrate the results obtained applying the analysis technique of Allegrini et al. (2002); Bohara et al. (2017) to the Physionet data of Peng et al. (1999). It is important to stress the different effects of different forms of meditation. Let us compare the results illustrated in Figure 7, Chi meditation, to those illustrated in Figure 8, Kundulini Yoga meditation. In both case there is a tendency for meditation to increase the IPL index μ. In the case of Chi meditation the only subject not changing μ is C7. For all the other subjects, δ moves from above the dashed line to below it. The change in μ goes from the minimal change, for C1 and C5, changing from μ = 2.47 to μ = 2.54 to the maximal change, for C2, changing from μ = 2.28 to μ = 2.54. For Kundalini Yoga meditation the minimal change is for Y1, from μ = 2.64 to μ = 2.68. The maximal change is for Y4, moving from μ = 2.66 to μ = 2.85.

Yoga meditation, in addition to increasing μ, has the surprising effect of significantly increasing ∈. Allegrini et al. (2002) conjectured that 1 − ∈ is an indicator of stress that will eventually result in heart failure. If this interpretation is valid, we would infer that yoga is an efficient method to reduce stress. Apparently Chi meditation is not as efficient in reducing stress as that of Kundulini Yoga, since for some individuals, *C*3, *C*4 and *C*8, ∈^2^ is decreasing rather than increasing.

**Figure 9:**
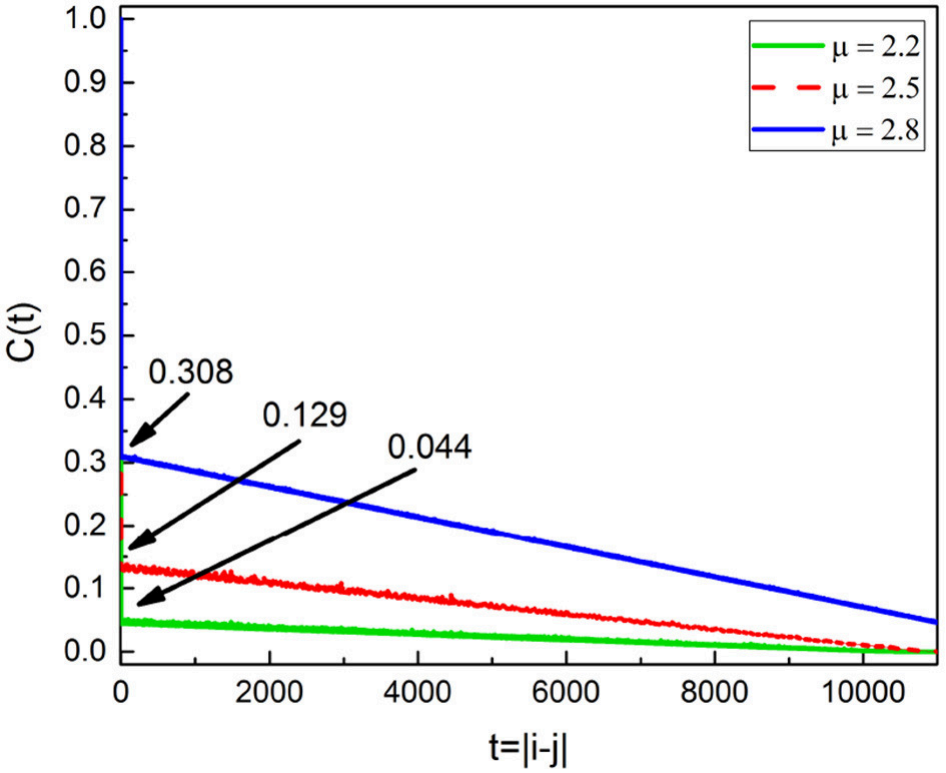
Correlation rate C(*t* = |*i* − *j*|) of Equation (4) for the subordinated oscillator with Ω = 0.8. From the top, the blue, red and green curves are for μ = 2.8, 2.5 and 2.2 respectively. As μ decreases C(1) decreases as labeled.

Actually this latter inference might be misleading and Chi meditation also has the effect of decreasing stress, although with less intensity than Kundulini Yoga meditation. As explained in earlier work (Bohara et al., 2017; Allegrini et al., 2002), the criterion adopted to evaluate ∈ is not an exact prescription and is based on the assumption that in the absence of Poisson events we have *C*(1) ≈ 1. As an effect of meditation-induced coherence this condition is violated and the value *C*(1) in the absence of Poisson events is much smaller than 1 and decreases as the IPL index μ comes closer to 2. This important property is illustrated in Figure 9. The adoption of a more precise way to evaluate ∈ may lead to the conclusion that Chi mediation also makes ∈ increase for all practioners.

## 5. Conclusions

In addition to the well known fact that meditation generates “exaggerated heart rate oscillations” (Peng et al., 1999) we find that meditation does not suppress the occurrence of crucial events. As pointed out in an earlier publication (Bohara et al., 2017) these crucial events are usually found embedded in a cloud of uncorrelated and irrelevant events, prompting us to introduce the notion of dressing. A dressed crucial events is one embedded in a cloud of Poisson events, whereas the time interval between bare crucial events is empty. The exaggerated heart rate oscillations are the form of dressing triggered by meditation. The adoption of the stripe technique of analysis (Bohara et al., 2017) made it possible to quantify the level of dressing by means of the measure ∈^2^. This enabled them to interpret 1 − ∈ as the percent of Poisson events affecting the HRV time series. Allegrini et al. (2002) conjectured that the Poisson events are generated by stress and that an excessive amount of such events as measured by ∈^2^, i.e., stress, may be the cause of heart failure. The results depicted in Figure 8 show that the Kundalini Yoga meditation yields, using the above interpretation, a significant reduction in the effect of stress. Consequently, this paper affords a technique of analysis of HRV that may be used to directly quantify the benefits of meditation, which are currently evaluated indirectly, through the observation of psychological health (Gard et al., 2014).

Gard et al. (2014) presented a very interesting discussion of the bottom-up and top-down mechanisms activated by yoga. We find that the meditation-enhanced cognition they discuss has the important effect of making the IPL index μ move from a region close to the border μ = 2, which corresponds to ideal 1/*f*-noise, see Equation (5), to a region closer to the border with the Gaussian basin of attraction, μ = 3 (Annunziato and Grigolini, 2000).

Does meditation contribute to making physiological processes more complex, or less complex? We posit the plausible conjecture that meditation makes physiology more complex (Sarkar and Barat, 2008; Bhaduri and Ghosh, 2016). The observation made herein that the heartbeat dynamics, as manifest in HRV time series, is a mixture of Laplace determinism and crucial events leads us to the conclusion that meditation does, in fact, decrease the complexity of HRV. The more pronounced meditation-induced coherence is an enhancement of Laplace determinism and the significant increase of the IPL index μ signifies a tendency to move toward the Gaussian basin of attraction. The increase of ∈^2^, with a significant reduction of the percentage of Poisson events, facilitates the creation of regular oscillations.

The transition from a region close to μ = 2 to one closer to μ = 3 is a departure from the ideal 1/*f*-noise and fits the observation made by psychologists (Dotov et al., 2017) that, in accordance with the phenomenological philosopher Heidegger (1962), cognition is activated by addressing a difficult task. This is in line with the observation made by Correll (2008) that the rapid response to a difficult social identification task often reveals unconscious bias; one measure of the task's difficulty being the deeper cognitive transition from 1/*f*- noise to white noise, corresponding to μ ≫ 1. Meditation, producing a transition from the ideal 1/*f*-noise condition to the Gaussian basin of attraction, which in the Poisson limit μ → ∞ yields white noise, is a sign of a difficult task that yields relaxation and stress reduction, while the difficult task of bypassing the unconscious bias may be a source of stress.

Note that phenomena such as dreamless deep sleep (Allegrini et al., 2015) and brain dynamics under anesthesia (Stramaglia et al., 2017) yield a departure from the criticality condition, and this is interpreted as favoring a lack of sensitivity to perturbation (Solovey et al., 2015) in line with other recent results (Krzemiński et al., 2017). According to Varela and his co-workers Buddhist philosophy, a source of meditation techniques similar to those analyzed in this paper, is of fundamental importance in addressing the ambitious issue of cognition (Varela et al., 2016). For this reason we believe that the present research approach may contribute to the progress of cognition science.

We found also that the long-term practice of meditation has the effect of making permanent the meditation-induced physiological changes, making the periodic bump in the spectrum become permanent.

## 6. Future directions

### 6.1. HRV dynamics

The nature of HRV dynamics is the object of lively debates (Glass, 2009; Sassi et al., 2009; Valenza et al., 2017). The question of whether or not the normal heart rate is chaotic (Glass, 2009) is leading investigators to refine the concept of *complexity variability* using the tools of non-linear dynamics (Valenza et al., 2017) and to restate the multifractal nature of heart rate variability (Sassi et al., 2009). We focus on the important role of *crucial events*, advocated in 2002 by Allegrini et al. (2002). More recently the authors of Bohara et al. (2017) and Mahmoodi et al. (2017a) proved that the occurrence of crucial events activates multifractal fluctuations, thereby raising the question of what is the origin of crucial events. Herein we rely on the validity of the hypothesis that crucial events are generated by the phenomenon of Self-Organized Temporal Criticality (SOTC) (Mahmoodi et al., 2017b). The subordination theory of section 2 makes it possible to simplify the much heavier computational approach to the self-organization of interacting units, each of which is characterized by periodicity. For this reason much of our future research work will be devoted to establishing the connection between subordination and SOTC.

### 6.2. Heart-brain communication

Rapid progress is currently being made in experimentally establishing the phase synchronization between different areas of the brain (Kitzbichler et al., 2009). Phase coupling has also been established in brain-heart communication (Pfurtscheller et al., 2017), suggesting that a central command may exist. The research of Ivanov and coworkers (Bartsch et al., 2014; Liu et al., 2015; Lin et al., 2016) are of importance to properly plan the future research direction of research based on the results presented herein.

**Figure 10:**
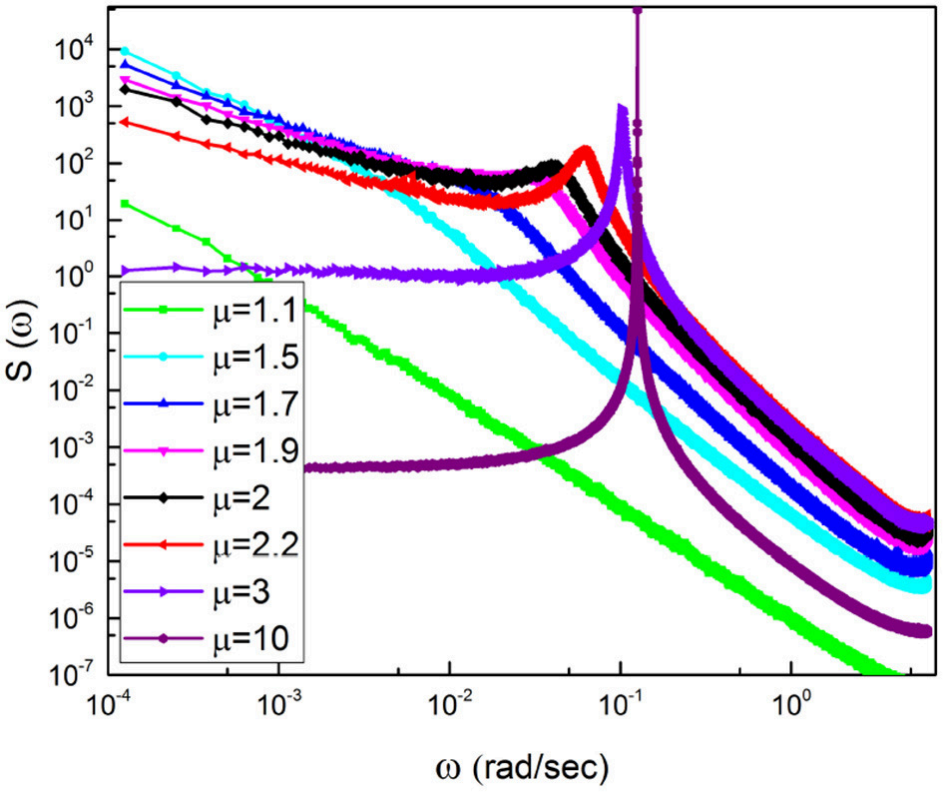
Spectra corresponding to cosine with Ω = 0.77 subordinated to an IPL PDF with a changing μ.

To explain why it is so, it is convenient to notice that according to the recent review paper of Mather and Thayer (Mather and Thayer, 2018) individuals with high HRV have better feelings of well-being than individuals with low HRV. This suggests a communication process with information moving from heart to several emotion regulating regions of the brain (Etkin et al., 2015), thereby implying breath control, an important ingredient of biofeedback techniques (Lehrer and Gevirtz, 2014), to be the source of therapeutic efficiency. Doubts have been raised regarding this interpretation, based on the conjectures that the enhanced oscillatory behavior may reflect “systemic fluctuations, rather than neuronal connectivity" (Tong et al., 2015). There is a connection between these doubts and the results of Liu et al. (2015) proving that the wake state corresponds to the largest number of neural links, which we conjecture to be a condition equivalent to criticality and to the generation of 1/*f* noise. Our results on the meditation-induced transition from values of μ close to the ideal condition μ = 2, generating 1/*f* noise, to μ = 3, the border with the Gaussian basin of attraction, are compatible with a significant intensity increase of coherent oscillations. Figures 2, 3 show some signs of this effect that is made much more evident by Figure 10.

Unfortunately no reliable theory yet exists to establish the true origin of this phase coupling. We are convinced that the subordination perspective rests on a process of self-organized temporal criticality (SOTC) (Mahmoodi et al., 2017b) and preliminary work has been completed that support this conjecture (Mahmoodi et al., unpublished).

It is interesting to notice that the adoption of subordination-SOTC perspective is expected to lead to results in line with other interesting findings (Lin et al., 2016) showing an alternate behavior from the brain leading heart to that of the heart leading the brain, in conflict with the impression that the emotion regulating process is based on information moving from heart to the brain.

The main result of the present paper that oscillations of increasing intensity are connected to moving from criticality to subcritical condition seems to be in conflict with the intuitive belief that meditation increases cognition, but it is not. Keep in mind that a distinction must be made between observations made during meditation and those made after mediation. Teper and Inzlicht (Teper and Inzlicht, 2013) find that meditators show a stronger executive control. This observation requires further theoretical scrutiny (Sedlmeier et al., 2018) and affords an incentive to move along the lines of our work with properly designed psychological experiments.

### 6.3. Breathing, meditation, and cognition

On the basis of results presented herein, we plan to address the problem of brain-heart communication experimentally to establish what effect meditation may have, if any, on any presumed action of the central command hypothesized by the authors of Pfurtscheller et al. (2017). The recent review (Mather and Thayer, 2018) that suggests “heart rate oscillations can enhance emotion by entraining brain rhythms in ways that enhance regulatory brain networks,” implies that the central command is exerted by the heart, whose regular oscillations are stimulated by the breathing process. This entails focusing our attention on Kundalini Yoga, since Kundalini Yoga rests on breathing control (Peng et al., 1999). We plan to analyze EEG with the same technique as that used in this paper to assess whether or not brain dynamics and heartbeats share the same restricted range of μ values. If they do, this would suggest modeling the transmission of information from heart to the brain and back using the theoretical tool of complexity matching (Mahmoodi et al., unpublished) based on the subordination-SOTC perspective used in this paper. We expect that the adoption of this theoretical approach may make it possible for us to explain the meditation-induced enhancement of alpha waves in accordance with the observation (Chandra et al., 2017) through the use of Kriya Yoga, which is different from Kundalini Yoga.

Kundalini Yoga is known to be an efficient way to treat certain psychiatric disorders (Shannahoff-Khalsa, 2004), but the therapists using this technique are looking for further enhancement of beneficial results (Khalsa et al., 2015). We anticipate that the adoption of the analysis techniques of this paper may facilitate the progress being made in this important field of research.

## Author contributions

All authors listed have made a substantial, direct and intellectual contribution to the work, and approved it for publication.

## Conflict of interest statement

The authors declare that the research was conducted in the absence of any commercial or financial relationships that could be construed as a potential conflict of interest.

## References

[B1] Allegrini P. Balocchi R. Chillemi S. Grigolini P. Palatella L. Raffaelli G. (2002). Short- and long-term statistical properties of heartbeat time-series in healthy and pathological subjects, in “*Medical Data Analysis,” Third International Symosium, ISMDA* (Berlin: Springer), 115.

[B2] Allegrini P. Grigolini P. Hamilton P. Palatella L. Raffaelli G. (2002). Memory beyond memory in heart beating, a sign of healthy physiological condition. Phys. Rev. E 65:041926. 10.1103/PhysRevE.65.041926 12005892

[B3] Allegrini P. Paradisi P. Menicucci D. Laurino M. Piarulli A. Gemignani A. (2015). Self-organized dynamical complexity in human wakefulness and sleep: different critical brain-activity feedback for conscious and unconscious states. Phys. Rev. E 92:032808. 10.1103/PhysRevE.92.032808 26465529 PMC4909144

[B4] Annunziato M. Grigolini P. (2000). Stochastic versus dynamic approach to Lévy statistics in the presence of an external perturbation. Phys. Lett. A 269, 31 10.1016/S0375-9601(00)00206-1

[B5] Ascolani G. Bologna M. Grigolini P. (2009). Subordination to periodic processes and synchronization. Physica A 388, 2727–2740. 10.1016/j.physa.2009.03.025

[B6] Bartsch P. R. Ivanov P. C. (2014). Coexisting forms of coupling and phase-transitions in physiological networks, in Nonlinear Dynamics of Electronic Systems. NDES 2014. Communications in Computer and Information Science, eds Mladenov V. M. Ivanov P.C Vol. 438 (Cham: Springer), 270–287.

[B7] Bhaduri A. Ghosh D. (2016). Quantitative assessment of heart rate dynamics during meditation: an ECG based study with multi-fractality and visibility graph. Front. Physiol. 7:44. 10.3389/fphys.2016.00044 26909045 PMC4754439

[B8] Bohara G. Lambert D. West B. J. Grigolini P. (2017). Crucial events, randomness, and multifractality in heartbeats. Phys. Rev. E 96:062216. 10.1103/PhysRevE.96.062216 29347370

[B9] Bradley R. T. McCraty R. Atkinson M. Tomasino D. Daugherty A. Arguelles L. (2010). Emotion Self-regulation, psychophysiological coherence, and test anxiety: results from an experiment using electrophysiological measures Appl. Psychophysiol. Biofeedback 35, 261–283. 10.1007/s10484-010-9134-x 20559707

[B10] Cakir R. Grigolini P. Krokhin A. (2006). Dynamical origin of memory and renewal. Phys. Rev. E 74:021108. 10.1103/PhysRevE.74.021108 17025394

[B11] Chandra S. Jaiswal A. K. Singh R. Jha D. Mittal A. P. (2017). Mental stress: neurophysiology and its regulation by Sudarshan Kriya Yoga. Int. J. Yoga 10, 67–72. 10.4103/0973-6131.205508 28546676 PMC5433115

[B12] Chow S. (2015). Meditation History. Available online at: https://www.news-medical.net/health/Meditation-History.aspx

[B13] Correll J. (2008). 1/f Noise and effort on implicit measures of bias. J. Pers. Soc. Psychol. 94, 48–59. 10.1037/0022-3514.94.1.48 18179317

[B14] Dotov D. Nie L. Wojcik K. Jinks A. Yu X. Chemero A. (2017). Cognitive and movement measures reflect the transition to presence-at-hand. New Ideas Psychol. 45:1 10.1016/j.newideapsych.2017.01.001

[B15] Etkin A. Büchel C. Gross J. J. (2015). The neural bases of emotion regulation. Nat. Rev. Neurosci. 16, 695–700. 10.1038/nrn4044 26481098

[B16] Feller W. (1971). An Introduction to Probability Theory and Its Applications, Vol. II, 2nd Edn. New York, NY: Wiley.

[B17] Gard T. Noggle J. J. Park C. L. Vago D. R. Wilson A. (2014). Potential self-regulatory mechanisms of yoga for psychological health. Front. Hum. Neurosci. 8:770. 10.3389/fnhum.2014.00770 25368562 PMC4179745

[B18] Glass L. (2009). Introduction to controversial topics in nonlinear science: is the normal heart rate chaotic? Chaos 19:028501. 10.1063/1.3156832 19566276

[B19] Goldberger A. L. Amaral L. Glass L. Hausdorff J. M. Ivanov P. C. Mark R. G.. (2000). PhysioBank, PhysioToolkit, and PhysioNet: components of a new research resource for complex physiologic signals. Circulation 101, e215–e220. 10.1161/01.CIR.101.23.e215 10851218

[B20] Graves T. Gramacy R. Watkins N. Franzke C. A (2017). Brief history of long memory: hurst, mandelbrot and the road to ARFIMA, 1951-1980. Entropy 19:437 10.3390/e19090437

[B21] Grigolini P. Palatella L. Raffaelli G. (2001). Asymmetric anomalous diffusion: and efficient way to detect memory in time series. Fractals 9, 439–449. 10.1142/S0218348X01000865

[B22] He B. J. Zempel J. M. Snyder A. Z. Raichle M. E. (2010). The temporal structures and functional significance of scale-free brain activity. Neuron 66, 353–369. 10.1016/j.neuron.2010.04.020 20471349 PMC2878725

[B23] Heidegger M. (1962). Being and Time, J. Macquarrie and E. Robinson, Trans. New York, NY: Harper Perennial.

[B24] Ivanov P. C. Nunes Amaral L. A. Goldberger A. L. Havlin S. Rosenblum M. G. Struzikk Z. R.. (1999). Multifractality in human heartbeat dynamics. Nature 399, 461–465. 10.1038/20924 10365957

[B25] Khalsa M. K. Greiner-Ferris J. M. Hofmann S. G. Khalsa S. B. S. (2015). Yoga-enhanced cognitive behavioral therapy (Y-CBT) for anxiety management: a pilot study. Clin. Psychol. Psychother. 22, 364-371. 10.1002/cpp.1902 24804619 PMC4224639

[B26] Kitzbichler M. G. Smith M. L. Christensen S. R. Bullmore E. (2009). Broadband criticality of human brain network synchronization. PLoS Comput. Biol. 5:e1000314. 10.1371/journal.pcbi.1000314 19300473 PMC2647739

[B27] Kou S. C. Xie X. S. (2004). Generalized langevin equation with fractional gaussian noise: subdiffusion within a single protein molecule. Phys. Rev. Lett. 93:180603. 10.1103/PhysRevLett.93.180603 15525146

[B28] Krzemiński D. Kamiński M. Marchewka A. Bola M. (2017). Breakdown of long-range temporal correlations in brain oscillations during general anesthesia. Neuroimage 159, 146–158. 10.1016/j.neuroimage.2017.07.047 28750775

[B29] Laplace P. S. (1951). A Philosophical Essay on Probabilities, translated into English from the original French 6th Edn., eds Truscott F. W. Emory F. L. New York, NY: Dover Publications.

[B30] Lehrer P. M. Gevirtz R. (2014). Heart rate variability biofeedback: How and why does it work? Front. Psychol. 5:756. 10.3389/fpsyg.2014.00756 25101026 PMC4104929

[B31] Lin A. Liu K. K. L. Bartsch R. P. Ivanov Pl. Ch. (2016). Delay-correlation landscape reveals characteristic time delays of brain rhythms and heart interactions. Philos. Trans. R. Soc. A 374:20150182. 10.1098/rsta.2015.0182 27044991 PMC4822443

[B32] Liu K. K. L. Bartsch R. P. Lin A. Mantegna R. N. Ivanov P. C. (2015). Plasticity of brain wave network interactions and evolution across physiologic states. Front. Neural Circ. 9:62. 10.3389/fncir.2015.00062 26578891 PMC4620446

[B33] Lukovic M. Grigolini P. (2008). Power spectra for both interrupted and perennial aging processes. J. Chem. Phys. 129:184102. 10.1063/1.3006051 19045381

[B34] Mahmoodi K. West B. J. Grigolini P. (2017a). On the dynamical foundation of multifractality. arXiv:1707.05988 [nlin.AO].

[B35] Mahmoodi K. West B. J. Grigolini P. (2017b). Self-organizing complex networks: individual versus global rules. Front. Physiol. 8:478. 10.3389/fphys.2017.00478 28736534 PMC5500654

[B36] Mandelbrot B. Van Ness J. (1968). Fractional Brownian motions, fractional noises and applications. SIAM Rev. 10, 422–437. 10.1137/1010093

[B37] Mandelbrot B. B. (1965). Time varying channels, 1/f noises, and the infrared catastrophe: or why does the low frequency energy sometimes seem infinite? in IEEE Communication Convention (Boulder, CO).

[B38] Mandelbrot B. (1967). Some noises with 1/*f* spectrum, a bridge between direct current and white noise. IEEEE Trans. Inform. Theory. 13, 289.

[B39] Mather M. Thayer J. F. G. (2018). How heart rate variability affects emotion regulation brain networks. Curr. Opin. Behav. Sci. 19, 98–104. 10.1016/j.cobeha.2017.12.017 29333483 PMC5761738

[B40] Montroll E. Weiss G. H. (1965). Random walks on lattices. II. J. Math. Phys. 6, 167 10.1063/1.1704269

[B41] Mori H. (1965). Transport, collective motion, and Brownian motion. Prog. Theor. Phys. 33, 423–455. 10.1143/PTP.33.423

[B42] Niemann M. Kantz H. Barkai E. (2013). Fluctuations of 1/*f*noise and the low-frequency cutoff paradox. Phys. Rev. Lett. 110:140603. 10.1103/PhysRevLett.110.140603 25166973

[B43] Peng C.-K. Mietus J. E. Hausdorff J. M. Havlin S. Stanley H. E. Goldberger A. L. (1993). Long-range anticorrelations and non-Gaussian behavior of the heartbeat. Phys. Rev. Lett. 70, 1343–1346. 10.1103/PhysRevLett.70.1343 10054352

[B44] Peng C.-K. Mietus J. E. Liu Y. Khalsa G. Douglas P. S. Benson H.. (1999). Exaggerated heart rate oscillations during two meditation techniques. Int. J. Cardiol. 70, 101–107. 10.1016/S0167-5273(99)00066-2 10454297

[B45] Peng C. K. Henry I. C. Mietus J. E. Hausdorff J. M. Khalsa G. Benson H.. (2004). Heart rate dynamics during three forms of meditation. Int. J. Cardiol. 95, 19–27. 10.1016/j.ijcard.2003.02.006 15159033

[B46] Pfurtscheller G. Schwerdtfeger A. R. Seither-Preisler A. Brunner C. Aigner C. S. Brito J.. (2017). Brain-heart communication: Evidence for “central pacemaker” oscillations with a dominant frequency at 0.1 Hz in the cingulum. Clin. Neurophysiol. 128, 183–193. 10.1016/j.clinph.2016.10.097 27912172

[B47] Sarkar A. Barat P. (2008). Effect of meditation on scaling behavior and complexity of human heart rate variability. Fractals 16, 199 10.1142/S0218348X08003983

[B48] Sassi R. Signorini M. G. Cerutti S. (2009). Multifractality and heart rate variability. Chaos 19:028507. 10.1063/1.3152223 19566282

[B49] Sedlmeier P. Lobe C. Quasten L. C. (2018). Psychological effects of meditation for healthy practitioners: and update. Mindfulness 9, 371–387. 10.1007/s12671-017-0780-4

[B50] Shannahoff-Khalsa D. S. (2004). An introduction to kundalini yoga meditation techniques that are specific for the treatment of psychiatric disorders. J. Altern. Complement. Med. 10, 91–101. 10.1089/107555304322849011 15025884

[B51] Shlesinger M. (2017). Origins and applications of the Montroll-Weiss continuous time random walk. Eur. Phys. J. B, 90, 93 10.1140/epjb/e2017-80008-9

[B52] Sokolov I. M. (2000). Lévy Flights from a continuous-time process. Phys. Rev. E 63:011104. 10.1103/PhysRevE.63.011104 11304231

[B53] Solovey G. Alonso L. M. Yanagawa T. Fujii N. Magnasco M. O. Cecchi G. A.. (2015). Loss of consciousness is associated with stabilization of cortical activity. J. Neurosci. 35, 10866–10877. 10.1523/JNEUROSCI.4895-14.2015 26224868 PMC4518057

[B54] Stanley H. E. Nunes Amaral L.A. Goldberger A. L. Havlin S. Ivanov P. Ch. Peng C.-K. (1999). Statistical physics and physiology: monofractal and multifractal approaches. Phys. A 270, 309–324. 11543220 10.1016/s0378-4371(99)00230-7

[B55] Stramaglia S. Pellicoro M. Angelini L. Amico E. Aerts H. Cortés J. M.. (2017). Ising model with conserved magnetization on the human connectome: Implications on the relation structure-function in wakefulness and anesthesia. Chaos 27:047407. 10.1063/1.4978999 28456159

[B56] Teper R. Inzlicht M. (2013). Meditation, mindfulness and executive control: the importance of emotional acceptance and brain-based performance monitoring. Soc. Cogn. Affect. Neurosci. 8, 85–92. 10.1093/scan/nss045 22507824 PMC3541488

[B57] Termsaithong T. Oku M. Aihara K. (2012). Dynamical coherence patterns in neural field model at criticality. Artif Life Rob. 17, 75–79. 10.1007/s10015-012-0020-x

[B58] Thiel F. Sokolov I. M. (2017). Time averages in continuous-time random walks. Phys. Rev. E 95:022108. 10.1103/PhysRevE.95.022108 28297905

[B59] Tong Y. Hocke L. M. Fan X. Janes A. C. deB Frederick B. (2015). Can apparent resting state connectivity arise from systematic fluctuations? Front. Hum. Neurosci. 9:285. 10.3389/fnhum.2015.00285 26029095 PMC4432665

[B60] Tuladhar R. Bologna M. Grigolini P. (2017). Non-Poisson renewal events and memory. Phys. Rev. E 96:042112. 10.1103/PhysRevE.96.042112 29347624

[B61] Valenza G. Citi L. Garcia R. G. Taylor J. N. Toschi N. Barbieri R. (2017). Complexity variability assessment of nonlinear time-varying cardiovascular control. Sci. Rep. 7:4279. 10.1038/srep42779 28218249 PMC5316947

[B62] Varela F. Thompson E. Rosch R. (2016). The Embodied Mind: Cognitive Science and Human Experience. Cambridge, MA: MIT Press.

[B63] Watkins N. W. (2016). Mandelbrot's 1/*f* fractional renewal models of 1963-67: the non-ergodic missing link between change points and long range dependence. *arXiv:1603.00738*.

[B64] Watkins N. W. (2017). On the continuing relevance of Mandelbrot's non-ergodic fractional renewal models of 1963 to 1967. Eur. Phys. J. B 90, 241 10.1140/epjb/e2017-80357-3

[B65] West B. J. (2013). Fractal Physiology and Chaos in Medicine, 2nd Edn. New Jersey, NJ: World Scientific.

